# Management of Complex Perianal Fistulas Using Platelet-Rich Plasma and Adipose-Derived Mesenchymal Stem Cells: A Case Series

**DOI:** 10.7759/cureus.77495

**Published:** 2025-01-15

**Authors:** Patroklos Goulas, Maria Karakwta, Alexandra-Eleftheria Menni, Apostolos Zatagias, Alexandros Zevgaridis, Natalia Valeria Pentara, Aristeidis Ioannidis, Stavros Panidis, Despoina Krokou, Nikolaos Gkouliaveras, Stylianos Apostolidis, Antonios Michalopoulos, George Koliakos, Vasileios Papadopoulos

**Affiliations:** 1 First Propaedeutic Department of Surgery, Aristotle University of Thessaloniki, AHEPA University Hospital, Thessaloniki, GRC; 2 Laboratory of Biological Chemistry, School of Medicine, Aristotle University of Thessaloniki, Thessaloniki, GRC; 3 Department of Surgery, Interbalkan Medical Center, Thessaloniki, GRC; 4 Department of Radiology, Aristotle University of Thessaloniki, AHEPA University Hospital, Thessaloniki, GRC; 5 Department of General Surgery, School of Medicine, Aristotle University of Thessaloniki, Thessaloniki, GRC

**Keywords:** case report series, complex perianal fistulas, experimental, mesenchymal stem cells, platelet-rich plasma (prp)

## Abstract

Perianal fistulas have posed a medical and surgical problem since ancient times. The plethora of surgical operations described nowadays for anal fistula treatment is real proof that there is no ideal therapeutic procedure. The cornerstone of all approaches is the equilibrium between the definitive treatment of the fistula with the maintenance of the anal continence mechanism, i.e., anal sphincters. Especially complex anal fistulas (multiple tracts, high transphincteric fistulas, rectovaginal and Crohn disease-associated anal fistulas) are difficult to deal with to preserve this therapeutic balance. Contemporary experimental procedures include the use of autologous biological products such as platelet-rich plasma (PRP) and mesenchymal stem cells (MSCs), either in their “raw” matrix (stromal vascular fraction, SVF) or after isolation and cultivation before delivered to the patient. Herein, we present a new experimental procedure that we implemented for four patients with perianal fistulas, which is safe and effective, easily reproduced, and cheap. It is a one-stage procedure that requires only simple means and could serve as a first-line approach in complex anal fistulas. In this article, we will present four indicative cases treated with this method, using different syringes for various patient conditions, while analyzing the corresponding clinical and imaging results.

## Introduction

Anal fistulas have been an eternal health problem, with difficult and painful therapeutic procedures even in the best hands. Since Hippokrates described his pioneering treatment with a cutting thread through the fistula tract (what was later meant to be the “seton” procedure), one could say that many things have changed, but all remain the same [1]. Ligation of intersphincteric fistula tract (LIFT), advancement flaps, ablation procedures (laser or radiofrequency devices), plugs, tissue glues, and human regenerative products (platelet-rich plasma (PRP), stromal vascular fraction (SVF), mesenchymal stem cells (MSCs)), all of them alone or in combinations are the latest proposals as perianal minimal invasive procedures [2]. They promise more continence security yet, but less definitive treatment without recurrence [2,3].

Concerning fistula classification, several systems have been proposed since Parks introduced his well-established, nowadays, classification system [4]. Whether clinical or radiological, all contemporary classification systems are based more or less on MRI imaging [5,6]. A common endpoint of these systems is guidance 11 for the surgeon in choosing the preferred surgical procedure, based not only on the course of the fistula tract but also on its complexity regarding the engagement of the sphincteric mechanism. [7,8].

Contemporary surgical approaches for complex anal fistulas still have the Seton method as a reference point, and many of the new ones would very much like to have the results of this method while also adding the best sphincter preservation. Even though new methods share the philosophy of the golden ratio between definitive treatment and anal continence, none of them has proven to be ideal. Although anal fistulas have been widely studied and various mechanisms of pathogenesis have been proposed, we still seem to be in shallow waters regarding their exact mechanisms of pathophysiology.

In this study, we presented four different cases of anal fistulae that have been treated by this method of PRPs.

## Case presentation

Materials and methods

PRP

A total of 20mL of whole blood was acquired up to five days preoperatively from the patients. After adding citrate-phosphate-dextrose solution (CPD), first centrifugation at 270g for seven minutes was done to collect the plasma, and then a second at 1000g for five minutes to collect the platelet fraction. After these steps, the supernatant was discarded, and the platelet fraction was placed at -80°C for 30 minutes to lyse the platelets and release the growth factors. After leaving the specimen at room temperature for some minutes, one final centrifugation at 1000g for five minutes was carried out, and the supernatant was collected. The final product, the PRP lysate, was stored in a refrigerator of -20°C until the surgical procedure [6].

Adipose-Derived MSCs

A tumescent liposuction from the abdominal wall was performed under general anesthesia. After infiltration of Klein’s solution (Klein’s solution contains lignocaine, epinephrine, and large amounts of saline) with a 1mm cannula, adipose tissue was harvested with a 3mm suction cannula. The fatty tissue collected was thoroughly washed and further processed with an inter-syringe procedure to emulsify the tissue. The specimen was centrifuged at 580g for 10 minutes, and the SVF with the cell pellet was at the bottom of the tube. The cell precipitate was previously evaluated and proved to have abundant viable mesenchymal cells [9]. The cells were collected and mixed with the PRP to be injected into the fistulous tract.

Procedure

The patient was placed in a supine position. Under general anesthesia, a liposuction procedure from the abdominal wall was carried out, lasting about 10 minutes and yielding around 100mL of adipose tissue. While the adipose tissue was then processed with the aforementioned procedure, the patient was placed in a lithotomy position. An endoscopy with an Eisenhammer anal retractor was done to inspect the internal orifice, the seton suture - if in place - was removed, and the fistula tract was thoroughly debrided with a curette or brush. After curettage, rinsing with saline followed, and the biomaterials to be injected were prepared. The internal opening of the fistula was closed with interrupted 2-0 Vicryl sutures (Ethicon, Inc., Bridgewater, USA), and the combination of PRP with stem cells was injected, half submucosally in the internal opening and half into the walls of the fistulous tract. The external opening, when identified and present, was left open. The patient was resuscitated in the operating room, and the whole procedure lasted about 95 minutes (mean time). All patients remained hospitalized for 24 hours and were discharged the next day with postoperative instructions consisting of the use of simple painkillers, normal anal hygiene, and follow-up at 7, 14, and 30 days. After that period, the follow-up was decided on an individualized basis, and all patients were re-examined at 6 and 12 months at least.

Evaluation

The postoperative Wexner score and postoperative complications were used to evaluate the procedure's safety. The efficacy of the method was evaluated based on the complete or near-complete healing of the fistula tract (absence of external opening or discharge). The minimum postoperative follow-up period was one year.

Case presentations of the patients

Four patients, two males and two females with complex anal fistulas, were treated with the above-mentioned procedure in an open-label study. A special written consent was acquired and the study protocol was approved by the Bioethics Committee of the Aristotle University of Thessaloniki (protocol number 370-9/10.5.2017). A Wexner incontinence score was calculated before and after the surgical procedure.

Case 1

**Figure 1:**
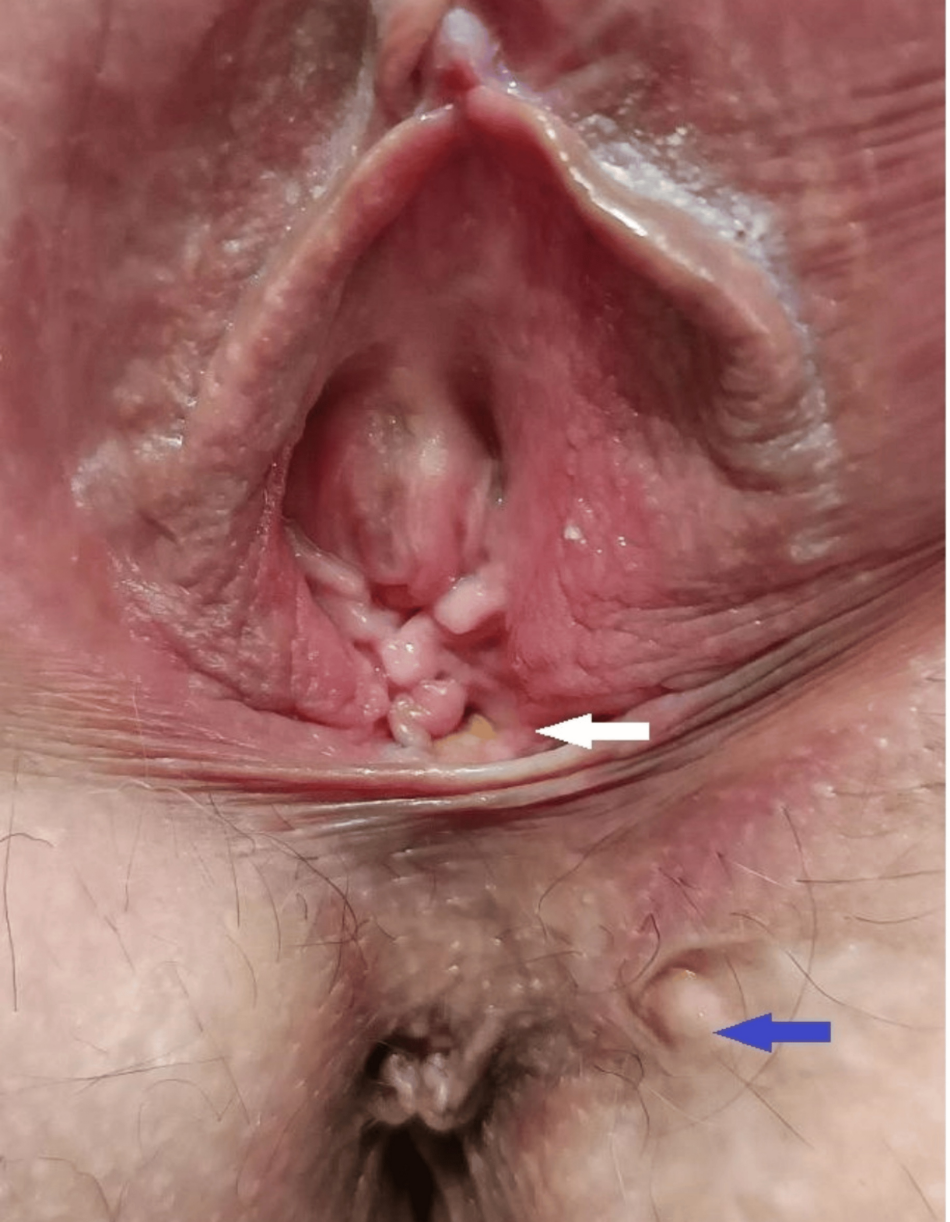
First patient with a complex rectovaginal fistula. The closed external orifice is marked with a blue arrow, and the second external orifice (vaginal) is marked with a white arrow. The internal orifice at the 12 o’clock position is not visible in the image.

The first patient was a 30-year-old woman who presented with a complex rectovaginal fistula with two external orifices, one at the vagina and one at the left perianal space, and one common internal orifice at 12 o’clock, having already had three surgical procedures for abscess drainage and seton placement. Her preoperative Wexner score was 10 (Figure 1).

Case 2

**Figure 2:**
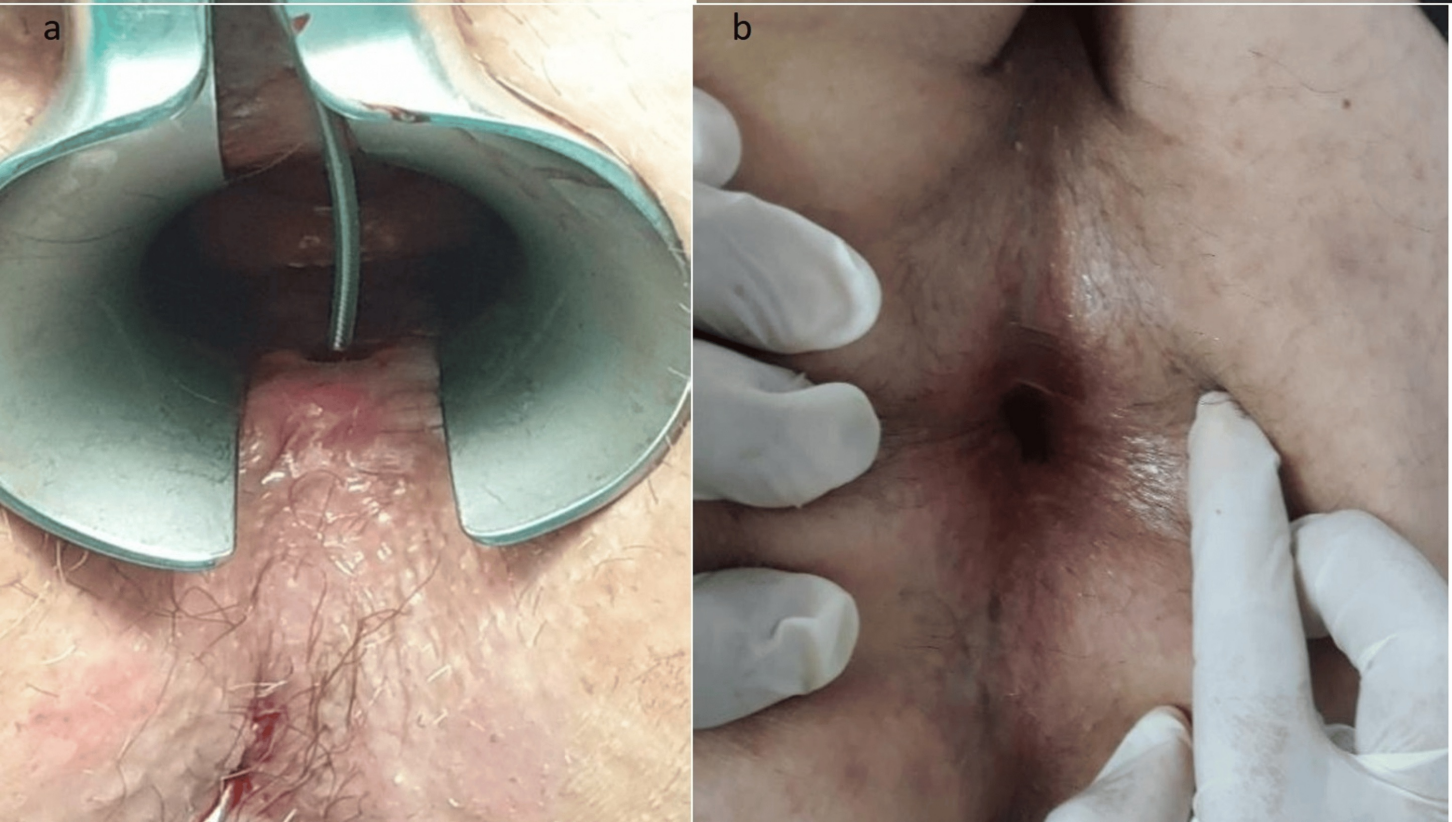
Second patient with a high transphincteric fistula. (a) Preoperative view; (b) Postoperative view

The second patient, a 65-year-old man, had two unsuccessful attempts at treating his high transphincteric fistula using tissue glue. His Wexner score before surgery was 5 (Figure 2).

Case 3

**Figure 3:**
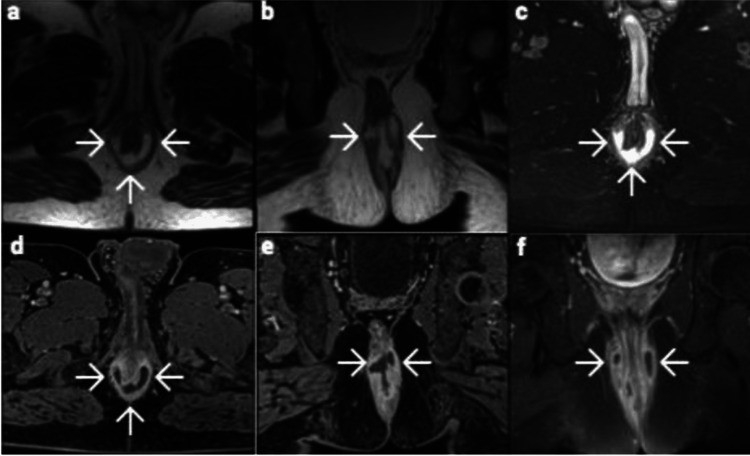
Third patient presenting with a perianal horseshoe fistula and an abscess. Magnetic resonance imaging findings: (a) Axial T2-weighted image (b) Coronal T2-weighted image (c) Axial fat-suppressed T2-weighted image (d) Contrast-enhanced fat-suppressed T1-weighted axial image (e) Contrast-enhanced fat-suppressed T1-weighted coronal image showing rim enhancement (f) Fat-suppressed T1-weighted coronal image

The third patient was a 45-year-old man with a complex horseshoe fistula with an abscess at the posterior wall of the anal canal, with no external opening and a history of multiple spontaneous or surgical drainages of the abscess into the anal canal, which has been left behind a complex horseshoe fistulous tract with an internal opening at 6 o’clock. His preoperative Wexner score was 4 (Figure 3).

Case 4

**Figure 4:**
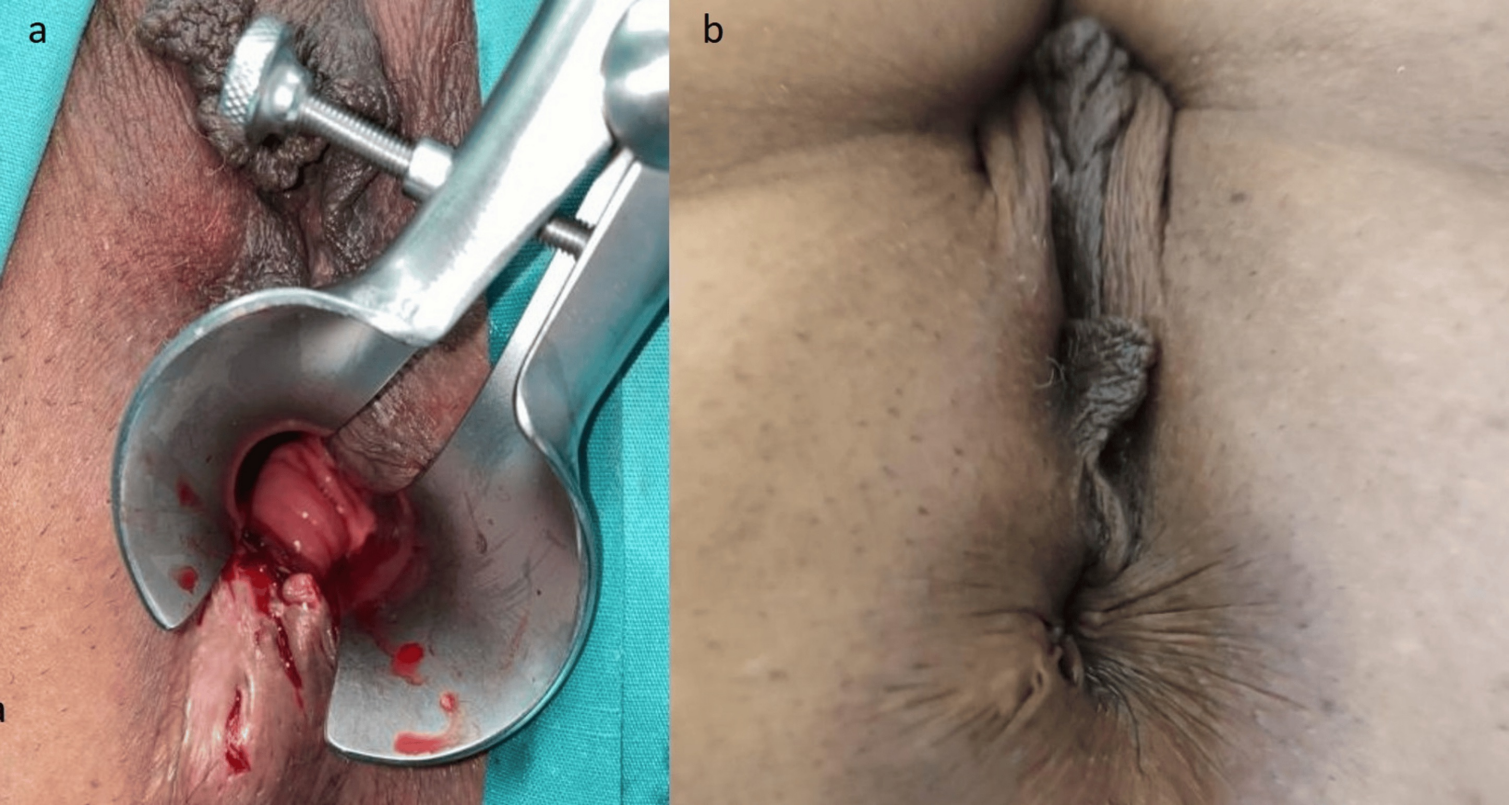
Fourth patient with perianal Crohn’s disease. (a) Preoperative view; (b) Postoperative view

Finally, a 40-year-old female with perianal Crohn's disease who was systematically receiving anti-tumor necrosis factor (anti-TNF) treatment was the fourth patient; nonetheless, her transphincteric fistulous tract was not responding to medication and required a seton placement. Her Wexner score prior to surgery was 3 (Figure 4).

Results

Both short- and long-term postoperative periods were uneventful for all patients. No perianal abscess, hematoma, or new fistula tract were observed. Post-operative pain was well-tolerated, and anal continence, measured with Wexner incontinence score, was not impaired by the operative procedure.

Case 1

During the first month postoperatively, there was only clear discharge from the two external orifices. Consecutively, the perianal external opening spontaneously closed, but the vaginal opening persisted, and in the second postoperative month, the discharge turned out to be mixed with fecal matter. After two and a half years of follow-up, the patient never had new episodes of inflammation or perianal abscess. The discharge of fecal matter is scarce, and her sexual life is normal. Her postoperative Wexner score improved to 6. Meanwhile, she had an uneventful course of pregnancy and gave birth to a healthy fetus by cesarean section with no deterioration of anal or sexual functions.

Case 2

Serous discharge from the external opening was seen during the first month following surgery, but it subsequently decreased. Spontaneous closure of the external opening was observed after three months. During the first postoperative year, he reported the spontaneous opening of the external orifice with minimum discharge twice, and he was subjected to the same procedure once more. After that, the external opening was re-epithelialized within three months, and he had no similar episodes during his two-year follow-up. Nowadays, he remains free of relapse or acute perianal episodes. His postoperative Wexner score improved to 2.

Case 3

The postoperative course was uneventful, with no discharge or relapse of perianal abscess. The patient had been previously subjected to multiple perianal abscess drainage procedures with a complex horseshoe abscess and fistula tract, refractory to surgical and medical treatment. After one year of follow-up, he reports no acute episodes and no anal discomfort being present before treatment. His postoperative Wexner score improved to 2.

Case 4

The seton was removed, and spontaneous healing of the fistula tract was observed during the first month after surgery. The external orifice closed completely, and within two years of follow-up, no relapse of the treated fistula occurred or new fistula due to her disease. Her postoperative Wexner score improved to 0.

All patients tolerated the surgical procedure well and reported an improvement in quality of life. Patients with complete healing and those with incomplete healing both replied that they would go through the surgical procedure again, as it improved their quality of life, and had never had a new acute episode of perianal abscess. Defecation and continence remained the same and improved, as proven by pre- and post-operative Wexner incontinence scores.

## Discussion

The exact pathophysiologic mechanism of forming a perianal fistulous tract is unknown. Concerning idiopathic cryptoglandular anal fistulas, the theory supporting the fistula creation depends on the hypothesis that an inflammation process begins within a gland of the anal crypts and expands unpredictably through the various structures of the surrounding anatomical area, including anal sphincters. Although this hypothesis is prominent even nowadays, there are still questions to be answered: what is the initiating signal to “light the fire” of perianal abscess and fistula? Why does this inflammatory process start, and what could predict the extent of the inflammation or the direction of the inflammatory tract? Numerous publications are trying to reach the truth - if there is much - about the etiology of this inflammation.

Regarding fistula etiology, there is a major division between those of cryptoglandular origin and those within the frame of a systematic disease (Crohn's disease, hidradenitis suppurativa) [8,9]. Both categories share an inflammatory background but have slightly different clinical manifestations, and the treatment of choice also differs. The role of bacteria has been extensively investigated yielding inconclusive results. Perhaps the study of Tozer et al. [9] summarizes this concern about the role of microbiota in perianal fistulous disease, pointing our attention elsewhere rather than to the bacteria that colonize the anus and perianal area [10,11]. Perianal Crohn's disease is considered to have a different pathophysiologic mechanism, starting an inflammation-mediated process from the anal canal epithelium and progressing with surrounding tissue erosion, but here also, the unanswered question of what is the “fire starter” remains. A genetic predisposition is also suspected for Crohn’s disease needing further investigation [12]. The tissue inflammation pathway is better studied in that patient’s category, showing the important role of lymphocytes, monocytes, dendritic cells, inflammatory cytokines, and matrix metalloproteinases. Our knowledge, according to the actions and interactions of the aforementioned cells, is progressing rapidly, but the way that the inflammatory process is regulated has to be further investigated. Nevertheless, MSCs have proven to be a part of this regulation mechanism by downregulating the inflammatory procedure and promoting healing via systematic and local pathways [13-15].

Some of the aforementioned procedures aim to heal the anal fistulas via an anatomic-mechanistic approach through the sealing of the internal opening and/or destroying or interrupting the fistulous pathway (LIFT, flaps, plugs, glues, ablation techniques). Others (PRP, stem cells, SVF) try more to interfere with the inflammation pathways by stopping the chronic inflammatory procedure and promoting healing, even though we have not yet completely understood these pathways [16,17].

Within this therapeutic labyrinth, we have implemented a surgical treatment combining modern procedures based on the administration of autologous PRP and adipose-derived MSCs. Within an open-label study, we follow a one-stage procedure that is safe, effective, and low-cost. Similarly to MSCs, PRP acts upon the regulation of the immune response when delivered locally through various growth factors that promote tissue healing [18-20]. The combination of MSCs and PRP seems to be a promising choice in our therapeutic armamentarium for perianal fistulizing disease as long as it is reserved for well-selected cases [21]. In our study, we implemented a simple method for collecting, manipulating, and isolating MSCs from the patient’s adipose tissue that requires very few technical means is easily reproducible, and yields a sufficient number of MSCs with very good viability. Combining the cells with autologous PRP, we enhanced the regenerative capacity of the administered MSCs.

Although the population of the study is tiny, these preliminary results - to be followed by larger study groups - are promising and consistent with similar studies [21,22]. The patients' long-term follow-up with durable results is also of great value, improving patients’ quality of life. It is important to point out that all patients’ quality of life improved after surgery, even if they had not achieved complete healing. Perhaps this was due to the persistent remission of their disease (minimum discharge) and the fact that none of them had a new acute episode, i.e., perianal abscess, during their long-term follow-up [23]. We could hypothesize that the administered MSCs with PRP acted in an anti-inflammatory way, and even though they failed to close the fistula in some cases, they did stop the chronic inflammatory process, preventing also an acute one. Another limitation of the study is the heterogeneity of the patients included, but this shows that MSCs and PRP could have a beneficial role in every perianal inflammatory process, irrespective of its origin. Finally, as ongoing research is evolving in the field of regenerative medicine and similar techniques are spreading widely, we could expect more studies with larger patient numbers and with randomized groups to interpret with more safety their results, although the standardization of similar techniques is difficult as every researcher uses his way of manipulating MSCs [24,25].

## Conclusions

Since ancient times, perianal fistulas have been a medical and surgical challenge. There is no ideal therapeutic approach, as evidenced by the multitude of surgical procedures that are reported today for the treatment of anal fistulas. The technique presented in this paper is a safe and easily applicable method for treating complex perianal fistulas. Although our study group is too small to produce solid results for the use of MSCs in perianal fistulas, the results collected so far are highly encouraging. However, more studies are needed to evaluate this method more properly.
